# Protocol for a phase I single-centre dose escalation trial of autologous thymus derived regulatory T cells in paediatric heart transplant recipients to prevent cardiac allograft vasculopathy (ATT-Heart)

**DOI:** 10.1136/bmjopen-2025-108683

**Published:** 2026-05-21

**Authors:** Apoorva Aiyengar, Giorgia Fanelli, Marco Romano, Prakash Patel, Fadi Issa, Dilveer Panesar, Nagarajan Muthialu, Abdel Douiri, Giovanna Lombardi, Michael Burch

**Affiliations:** 1 Great Ormond Street Hospital for Children, London, UK; 2 Peter Gorer Department of Immunobiology, King’s College London, London, UK; 3 Guy's and St Thomas’ NHS Foundation Trust, London, UK; 4 University of Oxford Nuffield Department of Surgical Sciences, Oxford, UK; 5 Paediatric Cardiothoracic Surgery, Great Ormond Street Hospital for Children, London, UK; 6 Department of Population Health Sciences, King’s College London, London, UK; 7 Institute of Cardiovascular Science, University College London, London, UK

**Keywords:** Paediatric cardiology, TRANSPLANT MEDICINE, Paediatric cardiac surgery

## Abstract

**Introduction:**

Cardiac allograft vasculopathy (CAV) is a critical predictor of the long-term success of heart transplantation and once it is established, progression to graft dysfunction and loss is inevitable, despite adherence to immunosuppression and medications that ameliorate cardiac risk factors. Regulatory T cells (Tregs) are key for maintaining immune balance in the periphery. Studies investigating adoptive transfer of ex vivo expanded Tregs isolated from blood have been shown to be feasible and safe with good evidence for Tregs reducing CAV lesions in animal models of transplantation. Here, we describe the protocol for the ATT-Heart Study which is a phase I clinical trial investigating autologous thymus-derived Treg cell therapy in nine paediatric heart transplant recipients.

**Methods and analysis:**

Patients will be recruited from the heart transplant waiting list at Great Ormond Street Hospital. Individualised autologous thymus-derived and expanded Tregs (TR006) will be injected into patients 3–6 months after transplant and follow-up will be conducted as per the post-transplant standard of care protocol with no wean of standard of care immunosuppression. Primary endpoint includes occurrence of Dose-limiting Toxicities in patients receiving TR006. Further data from blood tests, endomyocardial biopsy tissue, coronary imaging and clinical follow-up will be collected.

**Ethics and dissemination:**

This article is based on the ATT-Heart study Protocol (V.1.1; dated 19 December 2024). The ATT-Heart trial has received a favourable ethical opinion from the Health Research Authority and South-Central Oxford A Research Ethics Committee (IRAS Number: 1008875/REC reference: 24/SC/0333). Clinical trials authorisation approval from UK Medicines and Healthcare products Regulatory Agency has also been received. The clinical trial will be conducted in accordance with the principles of Good Clinical Practice and following the guidelines set as part of the Research Governance Framework for Health and Social Care and all applicable necessary local policies. It is intended that the findings of the clinical trial will be presented at national/international conferences and using social media and through patient groups for dissemination among their members. The results will also be published in international peer-reviewed journals.

**Trial registration number:**

ISRCTN15374803.

**STRENGTHS AND LIMITATIONS OF THIS STUDY**

- All eligible patients waitlisted for heart transplant (in one of the only two centres in the UK that perform paediatric heart transplant) will be approached and prospectively recruited and followed up in this study to minimise selection bias.
- As a phase I trial with primary focus on establishing safety of the cellular therapy at a range of doses, the study participants will not be randomised (or blinded) and results will not be able to quantify biological efficacy of Treg on cardiac allograft vasculopathy in a small population of patients with statistical significance.
- However, the immuno-monitoring schedule and analysis of data from patient biospecimens (collected before and after delivery of the therapy) will seek to study how the immune system of patients changes with cellular therapy administration to provide further information about the biological effect of the Tregs (which can be studied further in future studies).

## Introduction

### Study rationale

 The International Society of Heart and Lung Transplantation (ISHLT) registry data analysis shows that the median survival after heart transplantation in children across all age groups is just over 15 years, conditional on surviving the first year post-transplant when the risk of mortality is highest.^1^ Graft survival is longest in infant recipients (under 1 year of age with subsequent age groups showing a lower median survival rate). Early causes of mortality include graft loss, infection and acute rejection. However, 3 years post-transplantation, Cardiac Allograft Vasculopathy (CAV) is a leading cause of death.^1 2^ Similar trends are also seen in the Paediatric Heart Transplant Society database on heart transplants that took place in children between 1993 and 2010.^3^

CAV, also referred to as ‘transplant arteriosclerosis’ occurs as a result of endothelial dysfunction and inflammation due to an amalgamation of complex immunological and non-immunological factors. This ultimately results in activation of complement, cytokine production and proliferation of smooth muscle cells that causes accelerated thickening of the intimal layer of the coronary artery vessels of the donor heart.^4^ Once established, stenosis of the vessel lumen is diffuse making focal intervention at areas of stenosis impractical and ultimately futile for good long-term benefit.^5^,^7^

Progression of disease occurs despite optimisation of medication regimes to minimise rejection and control of ischaemic heart disease risk factors such as dyslipidaemia and hypertension, which is standard of care in heart transplant recipients, but these agents do not reverse established CAV lesions.^5 8^ Furthermore, denervation of the graft after transplant surgery can mask clinical symptoms of ischaemia due to interrupted blood supply to the graft and can also lead to an adverse cardiac event as a first clinical presentation.^4^

Following established CAV, graft survival is significantly reduced across all age groups with infants being worst affected with a median survival of 2 years after diagnosis.^1^ The only definitive treatment for CAV is re-transplantation, which has its own challenges and is not always a feasible strategy. With such serious implications and being a critical predictor of the long-term longevity of the transplanted organ, there is a pressing need to find a treatment for CAV.

Regulatory T cells (Tregs) are a subset of CD4^+^ T cells characterised by the constitutive expression of interleukin (IL)-2 receptor alpha chain (CD25^+^), low expression of IL-7 receptor alpha (CD127^-^) as well as high and stable expression of intracellular transcription factor Forkhead box P3 (FOXP3). The biological role of Tregs is to maintain immune homeostasis and prevent unwanted immune responses. Tregs exert their suppressive function, directly, via cell to cell contact or indirectly through the release of soluble factors.^9^,^12^ This immune modulating capacity of Tregs has been translated into a cell therapy and has been applied in the treatment of autoimmune diseases^13^,^16^ and in the prevention of solid organ transplant rejection^17^,^22^ and GvHD^23^,^26^

There is compelling evidence from numerous pre-clinical studies conducted by our group^27^ and others^28^,^35^ showing that adoptive transfer of Tregs reduced CAV and even promoted graft survival in mice that underwent heterotopic heart transplantation. In another study, immunodeficient mice transplanted with human vessels and reconstituted with allogeneic peripheral blood mononuclear cells (PBMCs) showed significant vascular intimal proliferation (ie, evidence of CAV lesions) and luminal narrowing; however, CAV was prevented in mice that were given concurrent PBMC and ex vivo expanded autologous human Tregs.^36^

### Dosing strategy

The dosing strategy has been developed based on data from a small selection of early phase trials across the world which have been conducted in both adult and paediatric cohorts (see online supplemental table 1 for reference). Autologous Treg therapy (derived from peripheral blood) has been studied in various autoimmune conditions (type 1 diabetes, multiple sclerosis and systemic lupus erythematosus). Specifically in the paediatric cohort, to our knowledge there is only one other study (NCT04924491) investigating thymus derived Tregs with dosing of up to 20×10^6^ cell/kg in infants up to 2 years old with no reported side effects in patients so far (trial is still ongoing).^21^ Our group (at KCL and GSTT) completed two clinical trials using in vitro expanded Tregs obtained from the blood of patients receiving either kidney (The ONE study^37^) or liver (ThRIL^20^) transplants. Altogether, 21 patients were treated with Tregs (up to 10×10^6^ cells/kg) with no safety concerns and some early signs of biological and clinical efficacy.

For ATT-Heart in children, our priority is confirming the safety of the doses in the first instance; hence, we have taken a conservative ascending dosing approach to dosing with two groups; the first cohort will receive 1–3×10^6^ cells/kg and if there are no serious side effects, the second cohort will receive 5–10×10^6^ cells/kg. The final cohort of patient will receive the highest tolerated dose (up to a maximum of 10×10^6^ cells/kg) from cohort two. As our trial will be recruiting children between the ages of 6 months and 16 years, some of our patients will be older, where age-related atrophy of the thymus tissue may also limit starting material for the manufacture of the Treg product, hence generating a wide dose range will also allow us to confirm the feasibility of our Treg expansion protocol. With the plans for immune-monitoring specified for our ATT-Heart trial, we hope to identify a dose which will have a biological effect that we can then test further in a phase II trial.

The evidence that Tregs can prevent CAV in preclinical models has been the basis for our new phase I clinical trial with Tregs in children receiving heart transplants. Most of the ongoing clinical trials have used Tregs purified from blood and then expanded in vitro. However, other sources of Tregs have previously been explored such as umbilical cord blood and thymus tissue.

The Tregs that will be injected in children receiving heart transplant in our trial (ATT-Heart) will be derived from the autologous discarded thymus and the protocol for their preparation will be presented in detail in the sections below. Patients will be recruited at Great Ormond Street Hospital (GOSH) and the Tregs will be prepared in the Good Manufacturing Practice (GMP) facility at Guy’s Hospital.

### Evidence from recent clinical trials with Tregs

Expanded Tregs have been used therapeutically in adult patients after renal^17 18^ and liver transplantation (ThRIL study).^20^ These clinical trials have shown the feasibility of the process, good safety profile and some signs of biological efficacy. In the ONE Study, an increase of regulatory B cells (Bregs) in the blood has been shown in renal transplant patients receiving Tregs compared with the control group^17 38^ and in analysis of their renal biopsies, focal immune lymphocytic infiltrates with a ‘regulatory’ signature was noted which was clinically distinct from ‘pro-inflammatory’ myeloid signature typically seen in biopsies of patients with rejection.^39^

The ThRIL study recorded a time-dependent, decreased donor reactive T cell responses in patients receiving Treg therapy while the response to third party remained unchanged.^20^ The success of the two clinical trials (ONE study and ThRIL) at King’s College Hospital with polyclonally expanded Tregs produced in the GMP facility at Guy’s Hospital, have paved the way for additional Treg trials in highly sensitised renal transplant patients; TWO study^40^ and GAMECHANgER-1 trial^41^ and also in patients with Crohn’s disease (TRIBUTE trial)^42^ and Aplastic Anaemia (TIARA) patients (*NCT05386264*).

In paediatric cohorts, in vitro expanded Tregs have been administered to children with a new diagnosis of type 1 diabetes in Poland (n=12)^14 15^ and in the Sanford Project T-rex study in the USA (n=64).^16^ No serious reactions related to the infusion were seen and these trials showed an increase in frequency of Tregs in blood following the infusion, with signs of biological efficacy by way of reduced need for exogenous insulin replacement in some children.^15^

The key difference between these trials and the ATT-Heart study is the origin of the Tregs for expansion. The above studies have used peripheral blood, whereas we plan to use the thymus tissue as starting material. The thymus is usually removed and discarded during open heart surgery. We and others have already shown that isolation and expansion of Tregs from the thymus is feasible^2143^,^45^ and more recently we have demonstrated that the protocol is translatable to the GMP facility.^46^

### ATT-Heart will use autologous thymus tissue from children as the source of Tregs

Having shown the safety of polyclonal Tregs in adults, we decided that the advantage of a therapy that can potentially allow minimisation of immunosuppression is greater in the paediatric population compared with adults. Furthermore, another advantage of Treg therapy in children receiving heart transplant is that the Tregs can be obtained directly from the thymus which is often routinely removed for operative reasons during open heart surgery. The thymus is a rich source of Tregs with excellent quality and suppressive ability that surpasses Tregs isolated from peripheral blood.^43^ This is particularly clinically relevant in paediatric trials as the smaller total blood volume in children limits the amount of safe blood draws required for adequate Treg extraction. The drug product developed for the ATT-Heart study will be named *TR006*.

We and others have published data on isolation and expansion of thymus derived Tregs (GMP compatible process) from thymus tissue obtained from paediatric donors following routine removal after cardiac surgery. We have shown that thymus derived Tregs are highly functional and stable even after cryopreservation.^45^,^47^ Furthermore, it is well known that the thymus is larger in infants and undergoes age related atrophy.

To our knowledge, only one other clinical study group is administering thymus derived Tregs in children which is currently ongoing in Spain^21^ (NCT04924491). Follow-up data from dosing of one infant patient demonstrated that the infusion was tolerated well with no adverse reactions and was able to repopulate the peripheral Treg frequency of the index patient to keep serial blood Treg values above the pre-transplant value throughout the follow-up period even after thymectomy and continuation of standard immunosuppression. In contrast, blood tests from the control cohort (n=4, did not receive Treg infusion) showed that Treg frequency dropped to below pre-transplant levels around 9 months post-transplant and dropped to 40% less than pre-transplant values at 2 years^21^ which is in keeping with previously published literature in children post-thymectomy and heart transplantation.^48 49^

**Figure 1:**
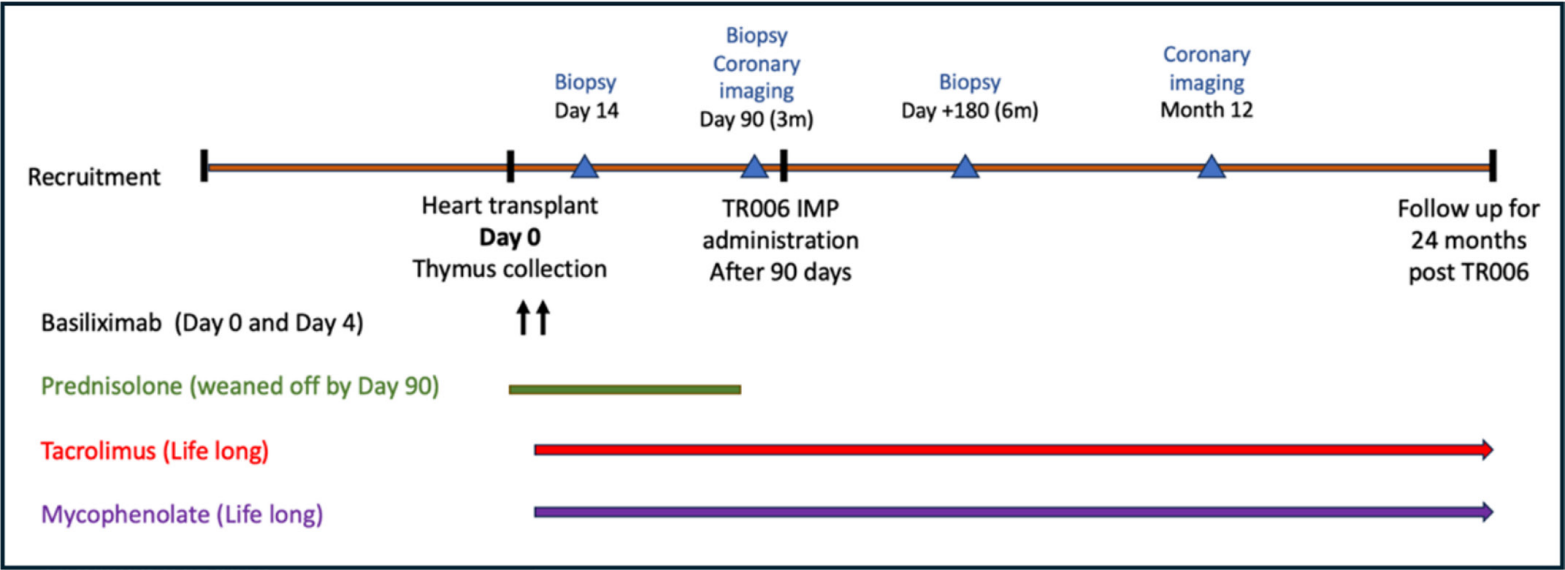
Timeline of key events in the ATT-Heart clinical trial, as well as the schedule for the immunosuppression given at GOSH. All patients undergoing heart transplant receive Basiliximab on the day of transplant and 4 days afterwards. Steroids are also commenced after heart transplantation and usual practice is to gradually wean down and stop steroids completely before the 3-month cardiac biopsy. Currently, tacrolimus and mycophenolate mofetil (MMF) are continued for life unless there are other clinical issues. GOSH, Great Ormond Street Hospital; IMP, investigational medicinal product.

In our institution, heart transplant patients receive induction therapy (the usual choice is Basiliximab which binds to the CD25 subunit of activated T lymphocytes). Pharmacokinetic studies in paediatric renal transplant patients receiving Basiliximab^50^ have shown that the CD25 blockade can be seen for up to 51.7 days. As Tregs constitutively express CD25 and this is a key marker for its regulatory function, we did not wish to administer the Treg containing product too soon after dosing with Basiliximab. Secondly, at the 3–6 month time point, the participant should have recovered after surgery and will also have had two routine surveillance biopsies (14 days and 3 months post-transplant as per figure 1) to confirm no signs of rejection, after which we can proceed with giving the Tregs. We will not be giving Tregs in any cases of suspected rejection as detailed in the exclusion criteria of our study.

### Aims and objectives of ATT-heart

#### Primary objective

- To determine the safety of administering a single dose of TR006 in nine children given at 3–6 months post-heart transplant in a planned dose escalation study design.

#### Secondary objectives

- To determine the feasibility of producing adequate cells in the investigational medicinal product (IMP) from the thymus of children in the study at the dose required per patient.
- To investigate the clinical responses to TR006 with regard to early CAV disease development, transplantation outcome and complications in the post-transplant journey for 24 months post-infusion.
- To determine the effect of TR006 on the immune system of the recipient, with a focus on the frequency of circulating Tregs and other T cell subsets, as well as changes within the transplant (immune monitoring data).
- To assess the feasibility of retaining participants for the duration of the study and their potential willingness to support future studies involving autologous Treg infusions.

## Methods and analysis

### Study design

This is a phase I, open label, single-centre clinical trial investigating two different dose banding ranges (1–3×10^6^ cells/kg and 5–10×10^6^ cells/kg) of autologous thymus derived Tregs (TR006) in nine paediatric heart transplant recipients. All clinical and patient-facing activity (including dosing) will occur at GOSH in London, UK.

**Figure 2:**
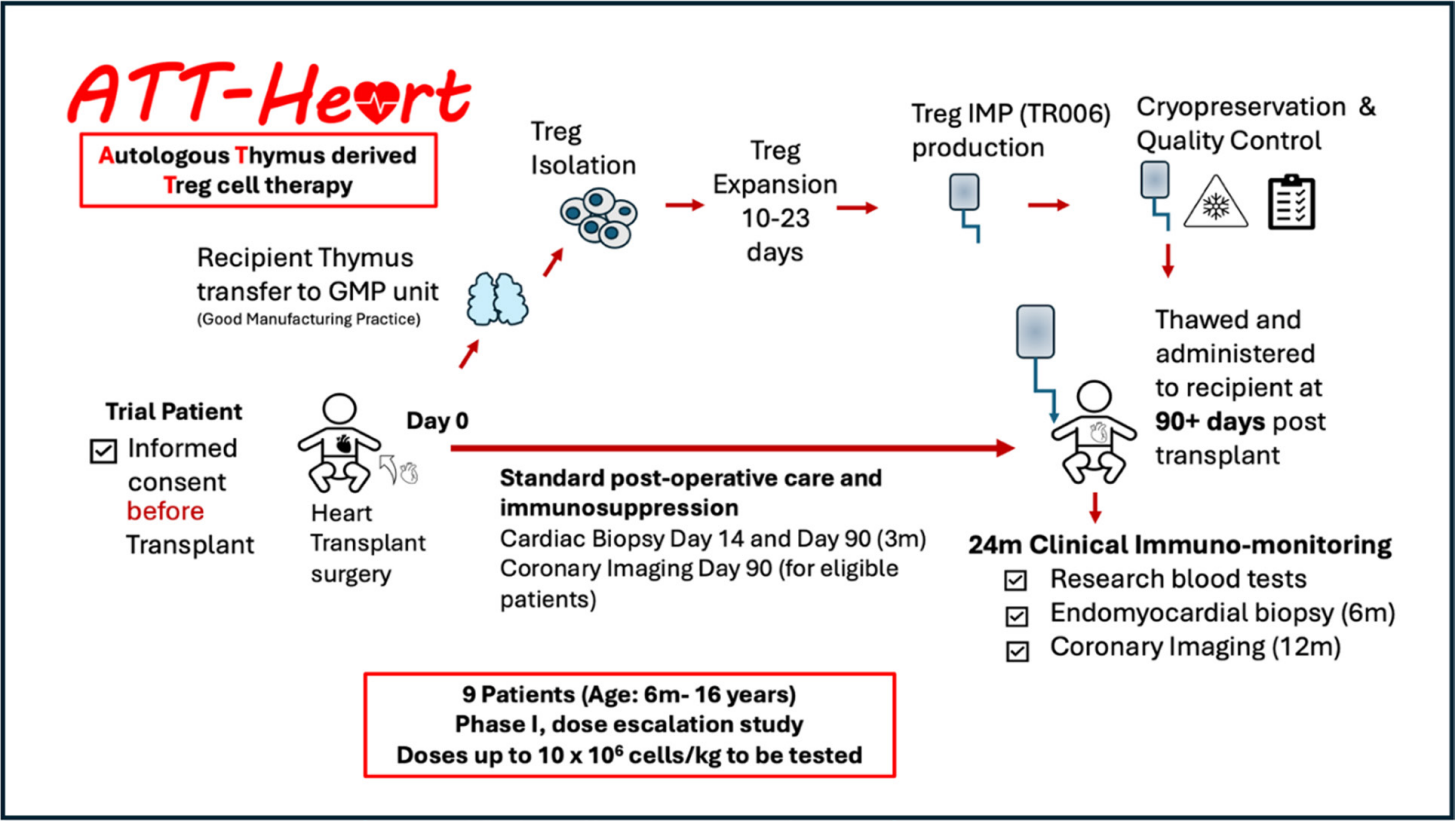
Schematic to highlight the key events in generating the investigational medicinal product (IMP) TR006 for ATT-Heart. IMP, Investigational Medicinal Product.

When a suitable organ is available for a recruited study patient (see figure 2), their thymus tissue will be collected during surgery. This tissue will be transported to the GMP Facility at Guy’s Hospital National Health Service (NHS) Foundation Trust and will form the starting material for the IMP production under GMP conditions, as outlined in the manufacturing section below.

Each child will progress through the routine post-operative care pathway (see figure 2). Standard of care immunosuppression will be maintained in all ATT-Heart trial patients and there are no plans to reduce it in this study.

Thymus tissue will be procured prior to the first dose of Basiliximab (anti-CD25 antibody that will affect Treg function) or other induction agent that might be given in theatre. Planned study procedures for ATT-Heart follow routine surveillance including endomyocardial biopsies at 14 days, 3 months and 6 months post-transplant in all patients. At 3 months, in addition to cardiac biopsy, coronary imaging (intravascular ultrasound (IVUS) or coronary angiogram) will be performed in children who weigh more than 25 kg. The outcome from the 3-month biopsy, particularly a negative result for histopathological signs of rejection, will be confirmed prior to administering TR006 to the patient.

Each study patient will receive a single dose of TR006 at 3–6 months post-transplant after initial baseline safety investigations are completed as per the study’s eligibility criteria. They will be monitored in hospital during and after the infusion for one night followed by further safety investigations prior to discharge the next day. Regular study visits as part of the safety monitoring stage will include investigations such as echocardiograms, 12 lead electrocardiograms and blood tests (clinical and research), before and after TR006 dosing. Please refer to onlinesupplemental tables 2  3 for details.

### Participant timeline

ATT-Heart participants will be reviewed and assessed throughout the trial from Screening to Safety Follow-up Month 24 (after TR006 dosing). Clinical blood tests will be performed throughout the duration of the study, and research blood samples will be collected up to the end of the study. Regular study visits will be scheduled up to Safety Follow-up Month 12 (after TR006 dosing) with one end of study visit scheduled after this (at Safety Follow-up Month 24). Please refer to onlinesupplemental tables 2  3 for more details.

### Study participant recruitment

It is the responsibility of the Principal Investigator or delegate at physician level (in line with GOSH consent policy) to obtain written informed consent from the patient’s parent/guardian (and where appropriate, also obtain assent from the participant) prior to performing any trial-related procedures. Please refer to the ATT-Heart Parents-Guardians Informed Consent Form and/or the ATT-Heart Parents-Guardians Thymus Collection Informed Consent Form within the online supplemental materials (and online supplemental file 2) for examples of the documents that will be used as part of the informed consent process.

The key inclusion and exclusion criteria for this study are listed in Box 1.

Box 1 **Key inclusion and exclusion criteria for the ATT-Heart clinical trial**

**Inclusion criteria**

- Male or female children aged between 6 months and 16 years.
- Children receiving a heart transplant.
- Single transplanted organ recipient.
- Willing and able to comply with the study visit schedule.

**Exclusion criteria**

- Active blood-borne viral and bacterial infection as specified in the study protocol.
- Age under 6 months or over 16 years at the time of heart transplant surgery.
- Multi-organ transplant.
- Previous recipient of any organ transplant.
- Highly sensitised patients at high risk of rejection.
- Participation in another interventional clinical trial of IMP during the study or within 28 days prior to the date of transplant (at the discretion of the Chief Investigator (CI).
- Allergy to any component/excipients used for the manufacture of the Treg product.
- History of previous sternotomy surgical procedure for congenital heart defect during which has had previous partial or full thymectomy.
- Diagnosis of congenital athymia due to DiGeorge syndrome.
- Female participant who is pregnant, lactating or planning pregnancy during the course of the trial.

Parents or carers of children who are listed for heart transplant at GOSH will be approached by the research team with written information about the study. Where possible, children will also be given information about the trial with age-appropriate patient information sheets to explain the study process. This may happen during their routine clinical appointment or at the time of transplant assessment with plans to obtain informed consent from potential participants (and their parents/carers) ahead of transplant surgery occurring. In certain cases, children on the heart transplant waiting list may require interim open-heart surgery by midline sternotomy for placement of a left ventricular assist device or commencing central mechanical support as a bridge to transplant; in which case thymus tissue may be procured at this stage.

Children who have had historical midline sternotomy procedures will be excluded from this trial as thymus tissue may have been previously resected.^51^ Furthermore, children with conditions such as DiGeorge syndrome may have T cell deficiency or be born with absent thymus will also be excluded from this trial.^52^

All efforts will be made to approach patients and carers who are eligible for this study prior to heart transplant surgery. In cases where transplant surgery has to happen in a short time frame for clinical reasons, interim consent to collect thymus tissue to commence manufacture of cell therapy will be sought from the parents/guardians during surgical consent before obtaining full consent at a later date to remain in the trial (to include TR006 administration and safety follow-up). This staged consent approach was incorporated into the study design on recommendation by the Research Ethics Committee in order to optimise the opportunity for patients requiring urgent transplantation surgery to enter this trial.

Typically CAV is a long-term complication of heart transplant; however, if there are signs of early CAV on the 3 month coronary imaging in a patient, we still plan to enrol them into the study and continue follow-up and coronary imaging at month 12 post-transplant as per the protocol. The presence and establishment of CAV post-transplant carries with it a poor prognosis as described in the study rationale.^1 5^

In such cases, if the patient chooses to continue, we may be able to determine the effect of TR006 on early CAV lesions on repeat coronary imaging. Furthermore, collection of clinical and immune-monitoring data in such a patient will help us track the disease process and determine how TR006 interacts with the patient’s immune system with signs of CAV. However, we would like to highlight that this is a phase I trial with a low starting dose of Tregs as our primary aim is to confirm safety of administration. As the dose required to achieve biological efficacy is yet to be determined, we cannot be certain of benefit for early CAV lesions with the dose of TR006 tested in this trial. This has been made clear in our patient information leaflet and consent forms and we will reiterate this in our counselling of future trials patients who do show early signs of CAV post-transplant before they continue in the trial.

### Donation declaration

Any organs/tissue used for ATT-Heart will be sourced ethically and any organs/tissue used will not have been sourced from executed prisoners or prisoners of conscience or other vulnerable groups.

In this study, the donated hearts are from deceased donors. The National Organ Retrieval Services, part of NHS Blood and Transplant, are a specialist team that will manage the procurement process, obtaining and preparing hearts for transplantation. This includes retrieval, characterisation, preservation and transport arrangements. The study team will not be involved in this process and it is independent of the study.

### Additional consent provisions for collection and use of participant data and biological specimens

Thymus tissue is usually routinely excised during heart transplant surgery and discarded. However, thymus tissue is rich in Tregs and hence can be considered a useful source for generation of the Treg product for the purpose of this study. The thymus tissue for ATT-Heart study participants, who consent to enter into the study, will be retained and transported to the GMP Facility to manufacture the Treg cell product (TR006).

ATT-Heart involves the collection and processing of biological specimens from participants (including blood and tissue samples from routine clinical procedures such as clinical blood tests and heart biopsy samples, as well as research samples).

Consent (and where appropriate, assent) will also be obtained for the retention and use of patient samples in related future research.

### Provision for participant care

Study participants will be considered as ‘live autologous thymus donors’, and will follow the same pathway as standard care heart transplant recipients before, during and after heart transplant surgery and thymus tissue procurement. Study visits have been designed to follow the standard post-heart transplant recipient care pathway.

ATT-Heart participants will attend GOSH for additional study visits (as TR006 dosing includes safety monitoring on the day of dosing and also an overnight stay to oversee the participant post-dose, and on Safety Follow-up Day 14 for more safety monitoring post-dose). There will also be remote safety monitoring carried out after dosing (as part of Safety Follow-up Days 2–13).

Team members will check at each visit with patients/parents/guardians that their consent to ATT-Heart is still valid. Patients will be presented with the option to continue to remain in the study, or withdraw, prior to IMP dosing and follow-up. Each participant has the right to withdraw from the trial at any time. Participants will continue on study until they express their wish to withdraw.

The participants and the parents/guardians of the participants who wish to withdraw from the study will be asked to confirm whether they are still willing to provide data collected as per routine clinical practice at visits. Their follow-up clinical care will be as per routine practice.

### TR006 manufacture

TR006 contains Tregs isolated from thymus tissue, which are polyclonally expanded in vitro, then administered as a single-use named patient therapy as an intravenous solution. As per our GMP compatible protocol,^46^ thymus tissue will first undergo mechanical digestion and then Treg isolation. This will require depletion of CD8^+^ cells followed by enrichment for CD25^+^ cells. The CD4^+^ CD25^+^ cell populations will be expanded for up to 23 days to obtain the Treg dose required for each patient. The polyclonal expansion will be undertaken in the presence of anti-CD3/CD28 beads, rapamycin and IL2.^46^

The final cell product will undergo a robust quality check assessment (immunophenotyping, potency, sterility testing, mycoplasma, endotoxin and viability testing) and the criteria to release for administration will in particular require ≥50% FOXP3^+^CD25^+^ expression of cells and >60% Treg suppressive ability at a 1:1 and 1:10 ratios of Tregs: T effectors. TR006 will remain cryopreserved for storage and delivery to GOSH. On the ward, the cell product will be handled by appropriately trained staff and thawed at the bedside prior to intravenous administration through a syringe pump by the research team.

### Intervention description: dose escalation strategy

Participants will receive a 10 mL single dose of TR006 at 1–3×10^6^ TR006/kg or at 5–10×10^6^ TR006/kg.

A single ascending dose design will be followed with groups of three patients in three cohorts. The first cohort of three patients will receive a lower dose of 1–3×10^6^ Tregs cells/kg and if no Dose-limiting Toxicities (DLTs) occur, then the second cohort (of three patients) will be given a higher dose of 5–10×10^6^ Treg cells/kg. The third cohort of three patients will receive the highest tolerated dose of Tregs from the second cohort (up to a maximum of 10×10^6^ Treg cells/kg).

These dose ranges were selected to match what we estimate to be the minimal anticipated biological effect doses based on the ThRIL and ONE study.^17 20^ Of note, the dose escalation schedule in ATT-Heart is more conservative than previous Treg trials conducted in children in Poland,^14^ the USA^16^ and Spain.^21^ However, as this is the first clinical trial in the UK with thymus derived Tregs, our priority is to determine safety of the IMP, as agreed with the Medicines Healthcare products Regulatory Agency (MHRA).

We will monitor biological activity of Tregs in vivo in this study. However, we plan to conduct further dose escalation and formally investigate biological efficacy in a future phase II trial if this trial confirms initial safety in patients and feasibility of manufacturing initial dose ranges.

### Provisions for post-trial care

All trial participants will be patients registered with GOSH for provision of their post-transplant care. Once the participant’s involvement in the study is over, they will continue to receive their usual post-transplant care (which includes lifelong medical follow-up).

### Study outcome measures

#### Primary endpoints

- The safety profile of the IMP is a key endpoint. DLTs such as presence of non-specific cytokine release, febrile episodes, hypersensitivity reactions, haematological derangement, biochemical imbalances and organ rejection that may occur up to 4 weeks post-IMP administration will be recorded. Clinically, these will be managed as per local guidelines. We will also record any suspected or confirmed DLTs that may occur beyond 4 weeks of IMP administration.

#### Secondary endpoints

- Feasibility of obtaining thymus tissue and expanding Tregs to an adequate dose (1–10×10^6^ cells/kg) as planned for the study will be documented for each patient entering the trial.
- To investigate the clinical responses to TR006, tests to determine the cardiac function, renal function and early CAV development (by recording the Minimal Intimal Thickness) by IVUS at protocol set time points. Furthermore, incidence of clinical morbidity events such as suspected or confirmed donor organ rejection, frequency of infection, hospitalisation events, immunosuppressive drug doses, transplantation outcome and mortality within 24 months of the IMP administration will be documented.
- To investigate the effect of TR006 on the immune system of the recipient, blood samples will be collected at different time points before and after TR006 infusion over the safety follow-up period. Brief details of the analysis techniques are outlined in table 1.

**Table 1:** Summary of immuno-monitoring analysis for ATT-Heart

Parameters measured	Method/test
Evaluation of circulating, proliferating, naïve and memory CD4^+^ and CD8^+^ T cell subsets and recent thymic emigrants in peripheral blood. Longitudinal comparisons will be conducted across different visits to assess the pharmacodynamic effects of TR006 based on the administered dose.	Flow cytometry
The deep phenotyping aims to comprehensively monitor 37 immune cell subsets and elucidate the modulatory effects of TR006 infusion on the patient’s immune landscape. Additionally, the study seeks to identify predictive immunological signatures associated with the early onset of CAV.	Mass cytometry (CyTOF)
Donor-specific antibodies: to assess risk of potential future antibody-mediated rejection.	Luminex
VEGF-C and A platelet factor 4: serum predictive factors for CAV development will be recorded.	ELISA
The assay of alloreactivity aims to measure T cell responses against donor antigens (and third-party antigens, as a control) to assess whether Treg therapy has specifically decreased the response against donor antigens, as previously published.^20^	Pleximmune test
Applying 5′ scRNA-seq to both the infused Treg product and the patients’ peripheral blood will enable clonal tracking and identification of cell therapy-derived populations over time.	Single cell RNA sequencing (scRNA sq)
Quantifying TH1/2/17 related cytokine serum levels to quantify cell-mediated or humoral-mediated immune responses to graft following TR006 infusion.	Multiplexed immunoassays (Meso Scale Discovery)
Histological signs of organ rejection in endomyocardial tissue biopsy by determining type of graft infiltration and inflammation.	(Standard of care) Immunohistochemistry staining
Assess composition and functionality of immune infiltrates and modulation of resident immune cells.	Spatial proteomic and transcriptomics

CAV, cardiac allograft vasculopathy; CyTOF, Cytometry by time of flight; VEGF, Vascular Endothelial Growth Factor.

In terms of the broader impact of our research, our clinical grade thymus derived Treg expansion protocol^45 46^ for producing functional and stable Treg therapy even after cryopreservation has been developed for this trial. As we plan to include children from a broad age range (6 months–16 years), the results from this trial may confirm feasibility of generating adequate doses with our Treg expansion protocol for older children and adolescents with smaller thymus tissue due to the expected age-related atrophy of thymus.

Producing Treg cellular therapy that can be cryopreserved and retain stability could be of benefit for a future ‘off the shelf therapy’. Clinical outcome data as well as immune-monitoring data from this trial will support identification of the dose at which biological efficacy is seen ahead of a phase II study in the paediatric heart transplant cohort. Patient and family reported data will also help shape a future trial design and conduct.

### Immuno-monitoring specific integrative data analysis plan

We will continue to prioritise Treg enrichment because our primary mechanistic questions are whether Tregs can be tracked by T Cell Receptor (TCR) clonality over time and how their phenotype and state changes post-infusion. To that end we will run 10× Chromium X 5’ captures on FACS-enriched Tregs with paired TCR VDJ. This preserves depth on a rare population and gives a robust clonotype map.

To provide the broader immune context, we will also perform whole-PBMC single cell (sc) RNA-sequencing with Cellular Indexing of Transcriptomes and Epitopes (CITE)-sequencing. We will employ cell hashing to multiplex samples and control cost. CITE-sequencing and TCR-sequencing will be generated from the same 10× capture so this approach will not compromise gene-expression depth on the enriched Treg runs. Further details on the time points are below.

We will use endomyocardial biopsies at day 14, month 3 and month 6 (ie, 3 months post-Treg infusion) and profile them by 10× Xenium. From each biopsy, we will focus on analysis of regions around small vessels to quantify endothelial activation, perivascular myeloid and lymphoid niches, fibroblast and matrix programmes and the presence of FOXP3+T cells. We will place control and Treg arm biopsies on the same slide to control for batch effects.

The primary clinical outcome is coronary allograft vasculopathy progression measured by IVUS (or coronary angiography in younger children) as the change in mean intimal thickness from month 3 to month 12, together with the prespecified CAV grade at month 12. Although CAV is defined in the coronary arteries, it is driven by immune and stromal processes that are reflected in the myocardial microvasculature. The spatial assay reads out those processes in situ. We will derive quantitative spatial features from the day 14, month 3 and month 6 biopsies, including endothelial activation scores, perivascular immune neighbourhood metrics and fibroblast remodelling signatures, and link them prospectively to the subsequent IVUS change between month 3 and month 12. We will aim to produce models that adjust for clinical covariates and integrate the circulating single cell RNA sequencing signals from the matched blood samples. This provides a biologically coherent bridge from tissue level mechanisms in myocardium to arterial remodelling in the coronaries. We will therefore test whether changes in spatial endothelial and perivascular signatures are linked to CAV progression, and whether these are modified in patients receiving TR006.

In short, the myocardial biopsies supply early spatial readouts of vascular inflammation and remodelling, the matched blood provides systemic context and Treg tracking, and both are linked prospectively to IVUS-based CAV progression between month 3 and month 12.

### Data collection, management and analysis

#### Plans for assessment and collection of outcomes

A study-specific ATT-Heart Data Management Plan, using electronic Case Report Forms (eCRFs), has been developed to describe the main processes and procedures used to ensure consistent and efficient collection and management of all data gathered for the clinical trial.

#### Data management

The ATT-Heart Data Management Plan has been created specifically for the clinical trial. Any study data will be entered in the eCRF platform, which automatically creates a protected audit trail for all data entries and changes.

The CI will act as custodian for the ATT-Heart study data.

Access to the eCRF platform will be password protected and electronic login credentials will only be issued to authorised individuals following the study-specific eCRF training.

#### Data analysis plan

Demographic data and baseline characteristics will be summarised to describe the populations analysed in this study (both all participants and participants administered at each dose level). Response will be reported descriptively based on the per-protocol population. Time to first response will be summarised by Kaplan-Meier curve. Median time to event estimation as well as the associated 95% CI will be reported. All other secondary endpoints will be summarised by rates and corresponding 95% CIs.

Interim safety data will be reviewed after dosing of the first and last patient of each cohort, with interim analyses provided to the Data Monitoring Committee (DMC) in order for decisions related to the next patient cohort to be made. This is particularly in relation to any safety concerns, or DLTs, prior to confirming progression of the dose escalation for the next cohort. In addition, the DMC will be informed of any Serious Adverse Reactions or Suspected Unexpected Serious Adverse Reactions (SUSARs) as they occur. All safety data will be analysed descriptively. Statistical comparisons between patient populations are unlikely to be used, but if needed, a 5% level of significance will be used.

Study data will be pseudo-anonymised and will be stored on a password protected computer at GOSH. Clinical data will be transcribed from the medical notes and source data sheets to the study-specific recording system. All data will be stored in line with UK General Data Protection Regulation and UK Data Protection Act 2018.

#### Clinical trial auditing

The Sponsor is responsible for implementing quality control and quality assurance.

Once study data are entered into the (trial-specific) ATT-Heart eCRF platform and saved, data validation checks will provide the first quality control step to check for completeness and plausibility of any manually‐entered trial data. These programmed edit checks will run online as soon as each data form is saved by the user.

The Sponsor Clinical Trial Co-ordinator will then perform source data verification (SDV) after the automatic validation checks have been completed. SDV will occur as part of monitoring visits to ensure the accuracy of manually entered trial data by the research team by comparing the eCRF data entry against the source data.

#### Harms/safety reporting

Adverse events, including Serious Adverse Events (SAEs) will be recorded from the Screening Visit to the Safety Follow-up Month 12 (after TR006 dosing) study visit. From the Month 12–24 safety follow-up period, only SUSARs will be reported. DLTs will be assessed from Week 0 to Week 4 of the study.

The Sponsor should report all the relevant safety information to the concerned Competent Authority (MHRA) and to the Research Ethics Committee concerned. The Sponsor shall inform all investigators concerned of relevant information about SUSARs that could adversely affect the safety of the study participants. The Sponsor will submit a development safety update report relating to this trial IMP to the MHRA and REC annually.

#### Confidentiality

In order to protect the privacy and identity of ATT-Heart participants, patients who take part in the study will be assigned a unique patient trial identifier on consent/assent (where appropriate). Only investigators and authorised staff at the study centre will be in possession of documents that link patient names to patient trial identifiers (eg, the signed informed assent/consent form(s) and ATT-Heart consent and enrolment log).

#### Access to data

The data used and/or analysed during this study are available from the CI (Professor Michael Burch) on reasonable request. For example, the full protocol and participant-level dataset will be available on request.

### Plans for collection, laboratory evaluation and storage of biological specimens for genetic or molecular analysis in this clinical trial/future use

All laboratory investigations that are required are part of the routine management of heart transplant patients (desirable assessments only required if being collected as part of routine clinical care), except the following additional research samples:

- PBMC panel (2 mL).
- Treg panel (1 mL).
- Circulating cytokines (3 mL).
- Alloreactivity (2 mL).
- Transcriptomic/gene expression (3 mL).

These will be collected at various study visits with a maximum of approximately 11 mL of additional blood per study visit collected from participants for the processing of research samples.

Some of the cardiac biopsy samples that are collected as per routine clinical care will be transferred to labs at GOSH and/or Guy’s Hospital and/or King’s College London for analysis and storage. Additionally, if there are any leftover clinical blood samples (collected as per routine clinical care), these also may be sent to labs at GOSH and/or Guy’s Hospital and/or King’s College London for analysis and storage.

If there are any cells remaining from the TR006 manufacturing process, these may be retained by the study team to further investigate the IMP.

Consent for optional samples will be sought from participants and their parents/guardians using the ATT-Heart Participant Information Sheet(s) and Informed Consent/Assent Form(s).

Samples will either be destroyed at the end of the study or may be used in future research studies with the participant’s consent.

### Clinical trial oversight

#### Trial management group

The trial management group (TMG) is led by Professor Michael Burch, the CI for this study. The group will include the CI, statisticians, clinical trial manager and representatives of the other teams involved in the delivery of the trial, including the GMP unit, laboratories and data management. The TMG will be responsible for the day-to-day management of the trial activities and will meet on a regular basis to discuss any trial related activities or issues.

#### Trial steering committee

The Trial steering committee (TSC) will provide advice for the conduct of the trial. It will comprise an Independent Chair and at least two other members. The TSC will provide overall supervision of the trial and ensure that it is being conducted in accordance with the principles of GCP and the relevant regulations. The TSC members will meet on an ad-hoc basis to discuss trial status, recruitment progress and any other relevant issues, and provide recommendations to the TMG/Sponsor.

#### Data monitoring committee

The DMC will be responsible for ongoing monitoring of the efficacy and safety of subjects in the study according to the DMC charter. The DMC charter will detail membership and terms of reference. The DMC members will be independent and supported by the CI, Trial Statistician and Clinical Trial Manager.

In order to ensure patient safety throughout the conduct of the trial, the DMC will review and evaluate accumulated safety data, study conduct and progress. The DMC will make the decisions about the continuation, modification or termination of the study. The recommendations made by the DMC to alter the conduct of the study will be forwarded to the Sponsor for a final decision. The Sponsor will forward such decisions to regulatory authorities, as appropriate.

Outside of the planned reviews, a DMC meeting could be triggered if specific safety events occur (as stated below):

- If a DLT is observed in two patients within a dosing cohort.
- If death of a patient infused with TR006 occurs.
- Or at any other time deemed necessary by the DMC chair.

#### Patient and public involvement

Patients and members of the public have been involved during the design and development of this trial. Paediatric recipients of heart transplants and their parents were consulted about the study topic and relevant age-appropriate literature. They provided detailed feedback (structure, content and wording) on the patient information leaflets, which was incorporated. Furthermore, trial rationale and design were presented to participants of the GOSH Young Persons and GOSH Parents and Carers Advisory Group (in two separate sessions), as well as members of an independent Research Ethics Committee. Positive feedback and enthusiasm for the project was received with valuable feedback on our trial design and patient-facing documentation.

We plan to maintain PPI during the trial by gathering responses to items in questionnaires or surveys exploring the study and treatment experience of the participants and their parents/guardians, which will help us assess the feasibility of retaining participants for the duration of the ATT-Heart study and willingness to join future trials (Secondary objective point 4). Additionally, members of the PPI group will form part of the Data Monitoring and TSCs for continual input during the conduct of the trial.

### Ethics approval and dissemination

The study protocol and related documents have been approved by an NHS Research Ethics Committee (REC), the Health Research Authority and the MHRA for Clinical Trial Authorisation. REC approval for the study was obtained from the South-Central Oxford A Research Ethics Committee on 19-DEC-2024 (REC Reference: 24/SC/0333).

The CI will submit a final report at the conclusion of the trial to the Sponsor and the necessary regulatory bodies within the stipulated timelines. Furthermore, it is intended that the results of the trial will be presented at international conferences and will be submitted for publication in a peer-reviewed scientific journal.

## Supplementary material

10.1136/bmjopen-2025-108683 online supplemental file 1

10.1136/bmjopen-2025-108683 online supplemental file 2

10.1136/bmjopen-2025-108683 online supplemental table 1

10.1136/bmjopen-2025-108683 online supplemental table 2

10.1136/bmjopen-2025-108683 online supplemental table 3
