# Supplementary material for: Protocol for a phase I single-centre dose escalation trial of autologous thymus derived regulatory T cells in paediatric heart transplant recipients to prevent cardiac allograft vasculopathy (ATT-Heart)
Source: BMJ Open. 2026 May 21;16(5):e108683. doi: 10.1136/bmjopen-2025-108683 (PMC13202142; doi:10.1136/bmjopen-2025-108683)
Supplement: online supplemental file 1 [file bmjopen-16-5-s001.docx]

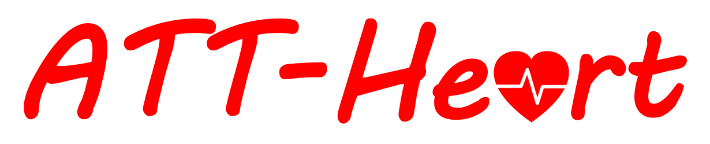


**ATT- Heart:**

An open label, single-centre dose escalation trial, investigating the safety and feasibility of **A**utologous **T**hymus derived regulatory **T** cell treatment for the prevention of cardiac allograft vasculopathy in children receiving **Heart** transplant.

**PARENTS / GUARDIANS INFORMED CONSENT FORM**

**Chief Investigator:** **Professor Michael Burch**

**Participant Study Identification Number:**

|  | Please **initial** box to indicate agreement. |
| --- | --- |
| 1. I confirm that I have received a personal copy of, and have read and understood, the Parents / Guardians Information Sheet dated _ _ - _ _ _ - _ _ _ _ (Version _ . _ ) for the ATT-Heart study. I have been given a copy of the Patient Information Sheet to keep. | [ ] |
| 1. I have been given sufficient time to discuss the ATT-Study with my child. | [ ] |
| 1. I am confident that my child realises that they have the choice to take part in the ATT-Heart study and that they understand what the study involves (to the best of their ability). | [ ] |
| 1. I have had the opportunity to ask questions about the ATT-Heart study, alongside my child, and we have had these answered satisfactorily. | [ ] |
| 1. I understand that my child’s participation is voluntary and that we are free to withdraw at any time without giving a reason, and without their medical care or legal rights being affected. | [ ] |
| 1. I give permission for the research team to access my child’s medical records for the purposes of this research study. | [ ] |
| 1. I understand that sections of any of my child’s medical notes and data collected during the study may be looked at by individuals from the Sponsor (Great Ormond Street Hospital), their representatives/agents, the regulatory authorities and individuals from the NHS Trust, where it is relevant to my child taking part in this research. I give permission for these individuals to have access to my child’s records which will include identifiable information. | [ ] |
| 1. I agree to my child’s General Practitioner being informed of my child’s participation in the study. | [ ] |
| 1. I agree to my child’s anonymous data being used for future ethically approved studies. | [ ] |

|  | Please **initial** box to indicate agreement. |
| --- | --- |
| 1. I understand that the information held and maintained by my child’s local NHS Trust may be used to help contact me or my child, or, provide information about my child’s health status by the study team. | [ ] |
| 1. I would like to receive a summarised version of the study results following the end of the study. | [ ] |
| 1. I agree for my child to take part in this study. | [ ] |
| 1. I consent to my child’s donated Treg cells, taken from their thymus, to being used for the manufacture of TR006. | [ ] |
| 1. I agree to the collection, processing, storage and analysis of any leftover manufactured TR006 product for research purposes as part of this study. | [ ] |
| 1. I understand that additional research blood samples will be collected from my child as part of this study and I agree for my child to provide these samples. | [ ] |
| 1. [OPTIONAL] I agree to the collection, processing, storage and analysis of any leftover clinical blood samples obtained from my child for research purposes as part of this study. | YES [ ]  NO [ ] |
| 1. [OPTIONAL] I agree to the collection, processing, storage and analysis of research blood samples obtained from my child for use in future ethically-approved research studies. | YES [ ]  NO [ ] |
| 1. I agree to the collection, processing, storage and analysis of clinical heart biopsy samples obtained from my child for research purposes as part of this study. | [ ] |
| 1. [OPTIONAL] I agree to my child’s pseudo-anonymised samples being used in ethically approved research in the future. I understand that this research may involve laboratories outside of the UK and EEA, including commercial partners. | YES [ ]  NO [ ] |

**Please feel free to use the below table write down any questions for the study team. You can also use this to make notes about the study.**

| **Questions / Notes:**  (From person with parental responsibility for the patient) | **Answers**  (From the Research Team) |
| --- | --- |
|  |  |

| Name of (child) patient: |  |
| --- | --- |

|  | _ _ - _ _ _ - _ _ _ _ |  |
| --- | --- | --- |
| Name of person with parental responsibility for the patient | Date  (DD - MMM - YYYY) | Signature |

Next section to be completed by the **person taking consent:**

|  | Please **initial** box to indicate agreement. |
| --- | --- |
| I have fully explained the purpose and nature (including benefits and risk) of this study to the participant and their parents/guardians in a way they can understand. I have invited them all to ask questions on any aspect of the study. | [ ] |
| I confirm that I have given a copy of the Parents / Guardians Participant Information Sheet and Informed Consent Form to the participant’s parents/guardians. | [ ] |

|  | _ _ - _ _ _ - _ _ _ _ |  |
| --- | --- | --- |
| Name of person taking consent. | Date  (DD - MMM - YYYY) | Signature |

|  | _ _ - _ _ _ - _ _ _ _ |  |
| --- | --- | --- |
| Name of witness (*if applicable*). | Date  (DD - MMM - YYYY) | Signature |

When completed: 1 copy for patient’s parents/guardians; 1 copy for medical notes; 1 (**original**) to be kept in Investigator Site File.
