# Supplementary material for: Protocol for a phase I single-centre dose escalation trial of autologous thymus derived regulatory T cells in paediatric heart transplant recipients to prevent cardiac allograft vasculopathy (ATT-Heart)
Source: BMJ Open. 2026 May 21;16(5):e108683. doi: 10.1136/bmjopen-2025-108683 (PMC13202142; doi:10.1136/bmjopen-2025-108683)
Supplement: online supplemental file 2 [file bmjopen-16-5-s002.docx]

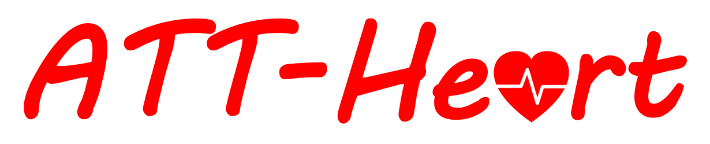


**ATT- Heart:**

An open label, single-centre dose escalation trial, investigating the safety and feasibility of **A**utologous **T**hymus derived regulatory **T** cell treatment for the prevention of cardiac allograft vasculopathy in children receiving **Heart** transplant.

**PARENTS / GUARDIANS THYMUS COLLECTION INFORMED CONSENT FORM**

**Chief Investigator:** **Professor Michael Burch**

**Participant Study Identification Number:**

|  | Please **initial** box to indicate agreement. |
| --- | --- |
| 1. I confirm that I have received a personal copy of, and have read and understood, the **Parents / Guardians Thymus Collection Patient Information Sheet** dated _ _ - _ _ _ - _ _ _ _ (Version _ . _ ) for the ATT-Heart study. I have been given a copy of the Short Patient Information Sheet to keep. | [ ] |
| 1. I have had the opportunity to ask questions about the ATT-Heart study, alongside my child if appropriate, and we have had these answered satisfactorily. | [ ] |
| 1. I understand that my child’s participation is voluntary and that we are free to withdraw at any time without giving a reason, and without their medical care or legal rights being affected. | [ ] |
| 1. I give permission for the research team to access my child’s medical records for the purposes of this research study. | [ ] |
| 1. I consent to my child’s donated Treg cells, taken from their thymus, to being used for the manufacture of TR006. | [ ] |
| 1. I agree to the collection, processing, storage and analysis of any leftover manufactured TR006 product for research purposes as part of this study. | [ ] |
| 1. I understand that additional research blood samples will be collected from my child as part of this study and I agree for my child to provide these samples. | [ ] |
| 1. I agree to the collection, processing, storage and analysis of clinical heart biopsy samples obtained from my child for research purposes as part of this study. | [ ] |
| 1. I understand that the research team will approach me later in order to give me more details about ATT-Heart and to obtain further consent to remain in the study. | [ ] |

**If you have any further questions, please use the next page to note these down and please also ask a member of the research team.**

**Please feel free to use the below table write down any questions for the study team. You can also use this to make notes about the study.**

| **Questions / Notes:**  (From person with parental responsibility for the patient) | **Answers**  (From the Research Team) |
| --- | --- |
|  |  |

| Name of (child) patient: |  |
| --- | --- |

|  | _ _ - _ _ _ - _ _ _ _ |  |
| --- | --- | --- |
| Name of person with parental responsibility for the patient | Date  (DD - MMM - YYYY) | Signature |

*Section to be completed by the person taking consent is on next page.*

Next section to be completed by the **person taking consent:**

|  | Please **initial** box to indicate agreement. |
| --- | --- |
| I have explained the purpose and nature (including benefits and risk) of this study to the participant and their parents/guardians in a way they can understand. I have invited them all to ask questions on any aspect of the study. | [ ] |
| I confirm that I have given a copy of the **Parents / Guardians Thymus Collection Participant Information Sheet** and **Thymus Collection Informed Consent Form** to the participant’s parents/guardians. | [ ] |
| I will provide the **ATT-Heart Parents / Guardians Participant Information Sheet** to the parents/guardians in due course so that they have the whole information about the study and can give full informed consent at a later date. | [ ] |

|  | _ _ - _ _ _ - _ _ _ _ |  |
| --- | --- | --- |
| Name of person taking consent. | Date  (DD - MMM - YYYY) | Signature |

|  | _ _ - _ _ _ - _ _ _ _ |  |
| --- | --- | --- |
| Name of witness (*if applicable*). | Date  (DD - MMM - YYYY) | Signature |

When completed: 1 copy for patient’s parents/guardians; 1 copy for medical notes; 1 (**original**) to be kept in Investigator Site File.
