# Supplementary material for: Protocol for a phase I single-centre dose escalation trial of autologous thymus derived regulatory T cells in paediatric heart transplant recipients to prevent cardiac allograft vasculopathy (ATT-Heart)
Source: BMJ Open. 2026 May 21;16(5):e108683. doi: 10.1136/bmjopen-2025-108683 (PMC13202142; doi:10.1136/bmjopen-2025-108683)
Supplement: online supplemental table 1 [file bmjopen-16-5-s003.docx]

**bmjopen-2025-108683 - Supplementary Material - Table 1**

**Supplementary Material Table 1:** Treg dosing strategies for adults and children published so far.

| **Reference/Study** | **Study Design & ID** | **Indication** | **Number of participants** | **Treg cell dose** | **SAEs** |
| --- | --- | --- | --- | --- | --- |
| Sanchez-Fuyeyo, 2020  (ThRIL trial) | Phase I  NCT02166177 | Liver transplantation | 9 | 1x10^6^/kg (n=3)  4.5x10^6^/kg (n=6) | 1 (infusion reaction) |
| Chandran, 2017 (TASK trial) | Phase I  NCT02088931 | Kidney transplantation | 3 | 4.5x10^6^/kg* (n=1)  4.6x10^6^/kg* (n=1)  5.2x10^6^/kg* (n=1) | 0 |
| Matthew, 2018 (TRACT trial) | Phase I  NCT02145325 | Kidney transplantation | 9 | 7.1x10^6^/kg (n=3)  14.3x10^6^/kg (n=3)  71.4x10^6^/kg (n=3) | 0 |
| Harden, 2020 (The ONE Study) | Phase I  NCT02129881 | Kidney transplantation | 12 | 1x10^6^/kg (n=3)  3x10^6^/kg (n=3)  6x10^6^/kg (n=3)  10x10^6^/kg (n=3) | 0 |
| Bluestone, 2015 | Phase I  NCT01210664 | Type 1 Diabetes | 14 | 0.7x10^6^/kg (n=3)  0.57x10^6^/kg (n=3)  4.6x10^6^/kg (n=4)  37x10^6^/kg (n=4) | 4 (metabolic related to diabetes, no infusion reactions or opportunistic infections) |
| Marek-Trzonkowska, 2012 | Phase I | Type 1 Diabetes, (Paediatric) | 10 | 10x10^6^/kg (n=4)  20x10^6^/kg (n=6) | 0 |
| Marek-Trzonkowska, 2014 | 1 year follow up of above study, plus 2 additional participants ISRCTN06128462 | Type 1 Diabetes, (Paediatric) | 12 | 10x10^6^/kg (n=3)  20x10^6^/kg (n=3)  30x10^6^/kg (n=6) | 0 |
| Dall’Era, 2019 | Phase I  NCT02428309 | Systemic lupus erythematosus | 1 | 1.4x10^6^/kg (n=1) | 0 |
| Chwojnicki, 2021 | Phase I EudraCT:2014-004320-22 | Multiple sclerosis | 14 | 40x10^6^/kg (n=11 IV)  1.0x10^6^/kg (n=3 IT) | 0 |
| Bender et al, 2024  (Sanford Project T-Rex) | Phase 2  Study, NCT02691247) | Type 1 Diabetes  (Paediatric) | 110 | 1-7x10^6^/kg (40)  11-24x10^6^/kg (24)  Placebo treatment (46) | 21  No SAEs were attributed as probably or definitely  related to the treatment. |
| [Esther Bernaldo-de-Quirós](https://pubmed.ncbi.nlm.nih.gov/?term=Bernaldo-de-Quir%C3%B3s+E&cauthor_id=37906166) et al, 2023 | Phase I/ II  EduraCT 2018-003574-28 | Heart transplantation  (Paediatric) | 10  (6 dosed so far) | 20 x 10^6^/kg | Ongoing  2 year follow up from 1 patient reported so far. No SAE |
