# Supplementary material for: Protocol for a phase I single-centre dose escalation trial of autologous thymus derived regulatory T cells in paediatric heart transplant recipients to prevent cardiac allograft vasculopathy (ATT-Heart)
Source: BMJ Open. 2026 May 21;16(5):e108683. doi: 10.1136/bmjopen-2025-108683 (PMC13202142; doi:10.1136/bmjopen-2025-108683)
Supplement: online supplemental table 3 [file bmjopen-16-5-s005.docx]

**bmjopen-2025-108683 - Supplementary Material - Table 3**

**Supplementary Material Table 3:** Summary of Trial Visits and Procedures after TR006 Dosing.

|  | **Safety Follow-up (after TR006 Dosing)** | | | | | | | | **End of Study** |
| --- | --- | --- | --- | --- | --- | --- | --- | --- | --- |
|  | Follow-up  Days 2-13 | Follow-up  Day  14 | Follow-up  Day  28 | Follow-up  Month  2 | Follow-up  Month  3 | Follow-up  Month  6 | Follow-up  Month  9 | Follow-up  Month  12 | Follow-up  Month  24 |
|  | +/- 2- Days | +/- 2- Days | +/- 2- Days | +/-  1 week | +/-  1 week | +/-  2 weeks | +/-  2 weeks | +/-  4 weeks | +/-  4 weeks |
| Informed Consent |  |  |  |  |  |  |  |  |  |
| Inclusion/Exclusion Criteria Review |  |  |  |  |  |  |  |  |  |
| Medical History |  |  |  |  |  |  |  |  |  |
| Concomitant Medication Review | X | X | X | X | X | X | X | X | X |
| Adverse Events Review | X | X | X | X | X | X | X | X | X |
| SUSAR Review | X | X | X | X | X | X | X | X | X |
| Remote Doctor Review | X |  |  |  |  |  |  |  |  |
| Doctor Review |  | X | X | X | X | X | X | X | X |
| Vital Signs |  | X | X | X | X | X | X | X | X |
| Height and Weight |  | X | X | X | X | X | X | X | X |
| 12 Lead ECG |  | X | X | X | X | X | X | X | X |
| Echocardiography |  | X | X | X | X | X | X | X | X |
| Intravascular Ultrasound |  |  |  |  |  |  | X ^e^ |  |  |
| Coronary Angiography |  |  |  |  |  |  | X ^e^ |  |  |
| Clinical Blood Tests ^b^ |  | X | X | X | X | X | X | X | X |
| Blood borne infection screen ^c^ |  |  |  | X |  |  |  |  |  |
| HLA Antibody Testing  (includes panel reactive antibodies and donor specific antibodies) |  |  |  |  | X |  | X |  | X |
| Urine Pregnancy Tests |  |  |  |  | X |  | X |  | X |
| Research Blood Samples ^d^ |  | X | X |  | X | X | X | X | X |
| Clinical Cardiac Biopsy |  |  |  |  | X |  |  |  |  |

| **Supplementary Material Table 3 Key** | |
| --- | --- |
| **Key** | **Description** |
| a | TR006 single dose to occur around 3 months post-cardiac transplant. Extra visit is required before if TR006 Dosing cannot be carried out within 30 days of the preceding study visit. Otherwise proceed to TR006 Dosing. |
| b | Haematology (FBC), Liver Profile (albumin, alkaline phosphatase, total bilirubin and ALT), Renal Profile (sodium, potassium, creatinine and urea), LDH and EBV and CMV viral load (where required). |
| c | HIV-1/2, HBsAg, HBC, HCV, HTLV, syphilis, Toxoplasmosis Gondii IgG, EBV IgG and CMV IgG (+ viral load where positive). |
| d | Research panel: Leukocyte subsets, peripheral Treg frequency, alloreactivity assay, circulating cytokines, transcriptomic/gene expression. Some/all of these tests will be done at marked visits. |
| e | Only for children over 25kg. |
| f | Clinical blood tests to additionally include testing for: C Reactive protein, ferritin, Creatinine Kinase, Bone profile, Serum Magnesium, Uric acid and coagulation screen. |
| g | Must occur within 24 hours of other procedures/tests performed as part of study visit. |
