# Supplementary material for: Protocol for a phase I single-centre dose escalation trial of autologous thymus derived regulatory T cells in paediatric heart transplant recipients to prevent cardiac allograft vasculopathy (ATT-Heart)
Source: BMJ Open. 2026 May 21;16(5):e108683. doi: 10.1136/bmjopen-2025-108683 (PMC13202142; doi:10.1136/bmjopen-2025-108683)
Supplement: Supplementary file 6 [file bmjopen-2025-108683.reviewer_comments.pdf]

PEER REVIEW HISTORY

BMJ Open publishes all reviews undertaken for accepted manuscripts. Reviewers are asked to complete a checklist review form and are provided with free text boxes to elaborate on their assessment. These free text comments are reproduced below.

ARTICLE DETAILS

Title (Provisional)

Protocol for a Phase I single-centre dose escalation trial of Autologous Thymic derived regulatory T cells in paediatric heart transplant recipients to prevent Cardiac Allograft Vasculopathy (ATT-Heart)

Authors

Aiyengar, Apoorva; Fanelli, Giorgia; Romano, Marco; Patel, Prakash; Issa, Fadi; Panesar, Dilveer; Muthialu, Nagarajan; Douiri, Abdel; Lombardi, Giovanna; Burch, Michael

---

VERSION 1 - REVIEW

**Reviewer**	**1**
**Name**	**Pradegan, Nicola**
**Affiliation**	**Padua University Hospital**
**Date**	**16-Oct-2025**
**COI**	**None**

I read with great interest this phase I trial protocol manuscript on a very original and emergin topic: in fact, authors present how they will deal with the administration of Treg cells in pediatric patients undergoing heart transplantation (obtained from autologous thymus) in order to define the safety of this therapy.

I only have few concerns which might be better explained in the text:

- first, why authors decided to start a similar trial first in the pediatric population rather than in adults. They should explain the rational for this choice.

- second: authors state that Treg will be adiminstered at 3-6mos, why they decided this specific timing? if a patient at 3mos already show initial signs of coronary lesions will you enroll the patient or not? what about patients with acute cell rejections or antibody mediated rejection?

- third: can you provide additional informations on how the cells will be obtained from the thymus and how they will be storaged until the infusion as well as the protocol to administer these therapy.

Thank you again for let me review this interesting manuscript.

**Reviewer**	**2**
**Name**	**Nubret, Karine**
**Affiliation**	**University Hospital Centre Bordeaux, Heart failure department, heart transplantation and MCS unit**
**Date**	**03-Nov-2025**
**COI**	**None**

This article presents a well-designed and promising protocol. Several aspects would benefit from clarification and it would be of interest to :

- provide a more detailed and explicit scientific rationale for the selected dosing regimen.

- develop the expected secondary endpoints to clarify the broader impact and relevance of the research

- describe in greater depth the integrative analysis strategy : how transcriptomic, phenotypic and functional datasets will be analyzed to obtain a comprehensive picture of the anticipated biological effects of TR006

VERSION 1 - AUTHOR RESPONSE

Reviewer: 1

Dr. Nicola Pradegan, Padua University Hospital Comments to the Author:

I read with great interest this phase I trial protocol manuscript on a very original and emerging topic: in fact, authors present how they will deal with the administration of Treg cells in pediatric patients undergoing heart transplantation (obtained from autologous thymus) in order to define the safety of this therapy.

I only have few concerns which might be better explained in the text:

- first, why authors decided to start a similar trial first in the pediatric population rather than in adults. They should explain the rational for this choice.

Thank you very much for your interest and feedback. Our group (at KCL and GSTT) has been involved in multiple early phase Treg trials undertaken in the adult population including the ONE Study, the TWO Study and GAMECHANgER Study (kidney Transplant patients) and ThRIL Study (liver transplant patients) which has demonstrated feasibility and safety of autologous Tregs (derived from peripheral blood) in adults. Following Treg therapy in two Treg trails in kidney transplant patients (ONE Study and TWO Study) minimization of immunosuppressive drugs has been successfully achieved (low dose TAC monotherapy).

Having shown the safety of blood-derived polyclonal Tregs, we decided that there are several advantages of a Treg therapy in children receiving heart transplants. First, the advantages of minimisation of immunosuppression are greater in the paediatric population compared to adults. Second, in children receiving heart transplants, Tregs can be obtained directly from the thymus (that is routinely removed and discarded during heart surgery for surgical reasons). We and others have shown that Tregs from the thymus are highly functional and more stable (compared to blood derived Tregs) even after cryopreservation (Romano et al, 2021: https://doi.org/10.1002/eji.202048949, Fanelli et al, 2025: https://doi.org/10.1186/s12967-025-06561-9, Bernaldo-de-Quiros et al, 2022: https://doi.org/10.3389/fimmu.2022.893576). Third, using Tregs from the thymus removes the need for drawing large blood volumes in children.

- Second: authors state that Treg will be adiminstered at 3-6mos, why they decided this specific timing? if a patient at 3mos already show initial signs of coronary lesions will you enroll the patient or not? what about patients with acute cell rejections or antibody mediated rejection?

In our institution, heart transplant patients receive induction therapy (usual choice is Basiliximab which is a CD25 binder). Pharmacokinetic studies in paediatric renal transplant patients receiving Basiliximab (Nagai et al, 2010: https://doi.org/10.5414/cpp48214) have shown that the CD25 blockade can be seen for up to 51.7 days. We decided to use the same clinical protocol for our trial and as Tregs constitutively express CD25 and this is a key marker for their regulatory function, we did not wish to administer the Treg containing product too soon after dosing with Basiliximab.

Secondly at the 3-6 month time point, the participant should have recovered after surgery and will also have had 2 routine surveillance biopsies (14 days and 3 months post-transplant as per figure 1B) to confirm no signs of rejection, after which can proceed with giving the Tregs. We will not be giving Tregs in any cases of suspected rejection (ACR or AMR) as detailed in the exclusion criteria of our study. This has been added to the manuscript in the "Introduction" section.

Typically, coronary allograft vasculopathy (CAV) is a long-term complication of heart transplant. However, if there are signs of early CAV on the 3-month coronary imaging in a patient, we still plan to enrol them into the study and continue follow up and coronary imaging at month 12 post-transplant as per the Protocol. The presence and establishment of CAV post-transplant carries with it a poor prognosis, as described in the study rationale (Rossano et al, 2017: https://doi.org/10.1016/j.healun.2017.07.018, Kindel, Pahl, 2012: https://doi.org/10.1111/j.1747-0803.2012.00666.x).

In such cases, if the patient chooses to continue, we may be able to determine the effect of Tregs (TR006) on early CAV lesions on repeat coronary imaging. Furthermore, collection of clinical and immune-monitoring data in such a patient will help us track the disease process and determine how TR006 interacts with the patient's immune system with signs of CAV.

However, we would like to highlight that this is a phase I trial with a low starting dose of Tregs as our primary aim is to confirm safety of administration. As the dose required to achieve biological efficacy is yet to be fully determined, we cannot be certain of benefit for early CAV lesions with the doses of TR006 tested in this trial. This has been made clear in our Patient Information Sheet(s) and Informed Consent Form(s) and we will reiterate this in our counselling of any study participants who do show early signs of CAV post-transplant before they continue in the trial.

- third: can you provide additional informations on how the cells will be obtained from the thymus and how they will be storaged until the infusion as well as the protocol to administer these therapy. Thank you again for let me review this interesting manuscript.

The section, "TR006 Manufacture," in the manuscript outlines the process of Treg isolation following mechanical digestion of thymus tissue and then immunomagnetic CD8 depletion and CD25 enrichment. Tregs are then expanded *in vitro* for a maximum of 23 days to reach the planned dose.

We have also briefly outlined our plans for quality control and checking of the cryopreserved product after thawing prior to release for a clinical batch. For brevity in the manuscript, we have not included the full manufacturing protocol.

Our group have recently published the GMP compatible Treg expansion protocol for production and storage of TR006 for the ATT-Heart Trial which has been referenced in the paragraph (Fanelli et al, 2025: https://doi.org/10.1186/s12967-025-06561-9)

Thank you very much for considering our manuscript and for your interest and feedback.

Reviewer: 2

Dr. Karine Nubret, University Hospital Centre Bordeaux Comments to the Author:

This article presents a well-designed and promising protocol. Several aspects would benefit from clarification and it would be of interest to :

- provide a more detailed and explicit scientific rationale for the selected dosing regimen.

Thank you for the feedback. We will add the following paragraph into the manuscript in a separate section titled ‘Dosing Strategy’. The summary table below can be added to supplementary section.

The dosing strategy has been developed based on data from a small selection of early phase trials across the world which have been conducted in both adult and paediatric cohorts (See table below for reference).

Autologous Treg therapy (derived from peripheral blood) has been studied in various autoimmune conditions (Type 1 Diabetes, Multiple Sclerosis and Systemic Lupus Erythematosus) in adult and in children. Specifically in the paediatric cohort, to our knowledge there is only one other study (ThyTech NCT04924491) investigating thymus derived Tregs with dosing of up to 20 x 10^6 cell/kg in infants up to 2 years old with no reported side effects in patients so far (and trial is still ongoing: Bernaldo-de-Quirós et al, 2023: https://doi.org/10.1084/jem.20231045).

Our group (at KCL and GSTT) completed two clinical trials using *in vitro* expanded Tregs obtained from the blood of patients receiving either kidney (The ONE study: Sawitzki et al, 2020: http://doi.org/10.1016/S0140-6736(20)30167-7) or liver (ThRIL: Sánchez-Fueyo et al, 2020: https://doi.org/10.1111/ajt.15700) transplants. Altogether, 21 patients were treated with Tregs (up to 10 x 10^6 cells/Kg) with no safety concerns and some early signs of biological and clinical efficacy. Two more clinical trials in renal transplant patients are underway (the TWO Study and GAMECHANgER).

For ATT-Heart (in children), our priority is confirming safety of the doses in the first instance, hence we have taken a conservative ascending dosing approach to dosing with 2 groups of patients; the first cohort will receive 1-3 x 10^6 cells/kg and if there are no serious side effects, the second cohort will receive 5-10 x 10^6 cells/kg. The final cohort of patients will receive the highest tolerated dose (max 10 x 10^6 cells/kg) from cohort two.

As our trial will be recruiting children between the ages of 6 months and 16 years, some of our patients will be older, where age related atrophy of the thymus tissue may also limit starting material for the manufacture of the Treg product, hence generating a wide dose range will also allow us to confirm feasibility of our Treg expansion protocol. With the plans for immuno-monitoring specified for our ATT-Heart trial, we hope to identify a dose which will have a biological effect that we can then test further in a Phase II trial.

**Reference/Study**	**Study Design & ID**	**Indication**	**Number of participants**	**Treg cell dose**	**SAEs**
Sanchez-Fuyeyo, 2020<br>(ThRIL trial)	Phase I<br>NCT02166177	Liver transplantation	9	1x10^6/kg (n=3)<br>4.5x10^6/kg (n=6)	1 (infusion reaction)
Chandran, 2017 (TASK trial)	Phase I<br>NCT02088931	Kidney transplantation	3	4.5x10^6/kg* (n=1)<br>4.6x10^6/kg* (n=1)	0

				$5.2x10^6$/kg* (n=1)	
Matthew, 2018 (TRACT trial)	Phase I NCT02145325	Kidney transplantation	9	$7.1x10^6$/kg (n=3) $14.3x10^6$/kg (n=3) $71.4x10^6$/kg (n=3)	0
Harden, 2020 (The ONE Study)	Phase I NCT02129881	Kidney transplantation	12	$1x10^6$ /kg (n=3) $3x10^6$ /kg (n=3) $6x10^6$ /kg (n=3) $10x10^6$/kg (n=3)	0
Bluestone, 2015	Phase I NCT01210664	Type 1 Diabetes	14	$0.7x10^6$/kg (n=3) $0.57x10^6$/kg (n=3) $4.6x10^6$/kg (n=4) $37x10^6$/kg (n=4)	4 (metabolic related to diabetes, no infusion reactions or opportunistic infections)
Marek-Trzonkowska, 2012	Phase I	Type 1 Diabetes, (Paediatric)	10	$10x10^6$/kg (n=4) $20x10^6$/kg (n=6)	0
Marek-Trzonkowska, 2014	1 year follow up of above study, plus 2 additional participants	Type 1 Diabetes, (Paediatric)	12	$10x10^6$/kg (n=3) $20x10^6$/kg (n=3)	0

	ISRCTN06128 462			$30x10^6$/kg (n=6)	
Dall'Era, 2019	Phase I<br><br>NCT02428309	Systemic lupus erythematosus	1	$1.4x10^6$/kg (n=1)	0
Chwojnicki, 2021	Phase I EudraCT:2014-004320-22	Multiple sclerosis	14	$40x10^6$/kg (n=11 IV)<br><br>$1.0x10^6$/kg (n=3 IT)	0
Bender et al, 2024<br><br>(Sanford Project T-Rex)	Phase 2<br><br>Study, NCT02691247)	Type 1<br><br>Diabetes<br><br>(Paediatric)	110	$1-7x10^6$/kg (40)<br><br>$11-24x10^6$/kg (24)<br><br>Placebo treatment (46)	21<br><br>No SAEs were attributed as probably or definitely<br><br>related to the treatment.
Esther Bernaldo-de-Quirós et al, 2023	Phase I/ II<br><br>EduraCT 2018-<br><br>003574-28	Heart transplantation<br><br>(Paediatric)	10<br><br>(6 dosed so far)	20 x 10^6/kg	Ongoing<br><br>2 year follow up from 1 patient reported so far.<br><br>No SAE

- develop the expected secondary endpoints to clarify the broader impact and relevance of the research

In the paragraph under secondary endpoints, the following detail will be added in to clarify the broader impact of our trial.

Our clinical grade thymic derived Treg expansion protocol for producing functional and stable Treg therapy even after cryopreservation has been developed for this trial. As we plan to include children from a broad age range (6 months- 16 years), the results from this trial may confirm feasibility of generating adequate doses with our Treg expansion protocol for older children and adolescents with smaller thymus tissue due to the expected age-related atrophy of thymus (Fanelli et al, 2025: https://doi.org/10.1186/s12967-025-06561-9, Romano et al, 2021: https://doi.org/10.1002/eji.202048949). Producing Treg cellular therapy that can be

cryopreserved and retain stability could be of benefit for a future 'off the shelf therapy' for children who lack a thymus for various clinical reasons.

Furthermore, this technology can also be translated to future trial designs. Clinical outcome data as well as immune-monitoring data from this trial will support identification of the dose at which biological efficacy is seen ahead of a Phase II study in the paediatric heart transplant cohort. Patient and family reported data will also help shape a future trial design and conduct.

- describe in greater depth the integrative analysis strategy : how transcriptomic, phenotypic and functional datasets will be analyzed to obtain a comprehensive picture
of the anticipated biological effects of TR006

A new section named, "Immuno-monitoring specific Integrative Data Analysis Plan," has been added to the manuscript outlining the following detail.

We will continue to prioritise Treg enrichment because our primary mechanistic questions are whether Tregs can be tracked by T Cell Receptor (TCR) clonality over time and how their phenotype and state changes post infusion. To that end, we will run 10x Chromium X 5' captures on FACS-enriched Tregs with paired TCR VDJ. This preserves depth on a rare population and gives a robust clonotype map.

However, as suggested, and to provide the broader immune context, we will also perform whole-PBMC scRNA-seq with CITE-seq. We will employ cell hashing to multiplex samples and control cost. CITE-seq and TCR-seq will be generated from the same 10x capture (feature barcodes and VDJ are separate libraries), so this approach will not compromise gene-expression depth on the enriched Treg runs. Further details on the time points are below.

We will use endomyocardial biopsies at day 14, month 3, and month 6 (i.e. 3 months post-Treg infusion) and profile them by 10x Xenium. From each biopsy we will focus on analysis of regions around small vessels to quantify endothelial activation, perivascular myeloid and lymphoid niches, fibroblast and matrix programmes, and the presence of FOXP3+ T cells. We will place control and Treg arm biopsies on the same slide to control for batch effects.

The primary clinical outcome is coronary allograft vasculopathy (CAV) progression measured by intravascular ultrasound (or coronary angiography in younger children) as the change in mean intimal thickness from month 3 to month 12, together with the prespecified CAV grade at month 12. Although CAV is defined in the coronary arteries, it is driven by immune and stromal processes that are reflected in the myocardial microvasculature. The spatial assay reads out those processes in situ. We will derive quantitative spatial features from the day 14, month 3, and month 6 biopsies, including endothelial activation scores, perivascular immune neighbourhood metrics and fibroblast

remodelling signatures, and link them prospectively to the subsequent IVUS change between month 3 and month 12.

We will aim to produce models that adjust for clinical covariates and integrate the circulating single cell signals from the matched blood samples. This provides a biologically coherent bridge from tissue level mechanisms in myocardium to arterial remodelling in the coronaries. We will therefore test whether changes in spatial endothelial and perivascular signatures are linked to CAV progression, and whether these are modified in the Treg-treated arm.

In short, the myocardial biopsies supply early spatial readouts of vascular inflammation and remodelling, the matched blood provides systemic context and Treg tracking, and both are linked prospectively to IVUS based CAV progression between month 3 and month 12.

We would be grateful for your consideration to publish this Protocol in BMJ Open.

VERSION 2 - REVIEW

**Reviewer**	**1**
**Name**	**Pradegan, Nicola**
**Affiliation**	**Padua University Hospital**
**Date**	**24-Jan-2026**
**COI**	

Thank you for the precise and deep explanation on my comments. I do not have any additional issues.
